# Swine influenza virus triggers ferroptosis in A549 cells to enhance virus replication

**DOI:** 10.1186/s12985-022-01825-y

**Published:** 2022-06-17

**Authors:** Jinghua Cheng, Jie Tao, Benqiang Li, Ying Shi, Huili Liu

**Affiliations:** 1 Institute of Animal Science and Veterinary Medicine, Shanghai Academy of Agricultural Science, Shanghai, 201106 People’s Republic of China; 2 Shanghai Key Laboratory of Agricultural Genetic Breeding, Shanghai, 201106 People’s Republic of China; 3 Shanghai Engineering Research Center of Pig Breeding, Shanghai, 201302 People’s Republic of China

**Keywords:** Swine influenza virus, Proteomics analysis, Ferroptosis, Iron disorder

## Abstract

**Background:**

Recently, Influenza A virus (IAV) has been shown to activate several programmed cell death pathways that play essential roles in host defense. Indeed, cell death caused by viral infection may be mediated by a mixed pattern of cell death instead of a certain single mode. Ferroptosis is a novel form of regulated cell death (RCD) that is mainly mediated by iron-dependent lipid peroxidation. Based on the proteomic data, we wondered whether IAV causes ferroptosis in host cells.

**Method:**

In this study, a quantitative proteomics approach based on an iTRAQ combined with LC–MS/MS was used to profile proteins expressed in A549 cells infected with H1N1 swine influenza virus (SIV). Meanwhile, we measured the intracellular iron content, reactive oxygen species (ROS) release and lipid peroxidation in response to SIV infection. Finally, a drug experiment was conducted to investigate the effects of ferroptosis on modulating SIV survival.

**Results:**

The bioinformatics analysis revealed several proteins closely relevant to iron homeostasis and transport, and the ferroptosis signaling pathway are highly enriched in response to SIV infection. In our experiment, aberrant expression of iron-binding proteins disrupted labile iron uptake and storage after SIV infection. Meanwhile, SIV infection inhibited system the Xc^−^/GPX4 axis resulting in GSH depletion and the accumulation of lipid peroxidation products. Notably, cell death caused by SIV as a result of iron-dependent lipid peroxidation can be partially rescued by ferroptosis inhibitor. Additionally, blockade of the ferroptotic pathway by ferrostatin-1 (Fer-1) treatment decreased viral titers and inflammatory response.

**Conclusions:**

This study revealed a new mode of cell death induced by IAV infection, and our findings might improve the understanding of the underlying mechanism involved in the interaction of virus and host cells.

**Supplementary Information:**

The online version contains supplementary material available at 10.1186/s12985-022-01825-y.

## Background

Influenza A virus (IAV) is a negative-sense RNA virus in the *Orthomyxoviridae* family. Swine influenza (SI) is caused by IAV and is an important acute respiratory disease in the swine industry. The major strains found in swine herds are the H1N1, H1N2 and H3N2 subtypes. Pigs are often considered as a “mixing vessel” for the generation of reassorted influenza viruses because they are highly susceptible to human and avian influenza virus infection [1]. The pathogenicity of influenza viruses is determined by many factors, and IAV have evolved strategies that modulate host responses to facilitate their propagation and infectivity [2, 3].

Cell death is crucial for various physiological processes in multicellular organisms. Ferroptosis is a novel form of regulated cell death (RCD) that is mainly mediated by iron-dependent lipid peroxidation. Occurrence of ferroptosis is characterized by the elevated levels of iron and phospholipid peroxides [4]. Excessive iron promotes reactive oxygen species (ROS) generation through the Fenton reaction and accelerates lipid peroxidation [5]. The glutathione peroxidase 4 (GPX4) and system Xc^−^ pathways are considered to be the primary signaling pathways associated with ferroptosis [6]. GPX4 reduces the lipid peroxidation rate at the expense of antioxidant glutathione (GSH), preventing cells from undergoing ferroptosis. System Xc^−^ is an amino acid antiporter that mediates the exchange of extracellular cystine and intracellular glutamate across the plasma membrane, which is an important process for GSH synthesis. Inhibition of system Xc^−^ induces ferroptosis through cysteine deprivation, subsequent GSH depletion, and ultimately inactivation of GPX4 [7]. One of the many factors that modulates cell death is viral infection. However, research on ferroptosis mediating the viral response is scarce, and whether IAV can induce ferroptosis is still unknown. In addition, few studies have investigated the role of ferroptosis in modulating virus survival.

Previous studies have shown that IAV infection can cause multiple forms of cell death, and the molecular mechanisms driving these pathways vary. The best-studied mechanism of IAV-induced cell death is apoptosis, which has been reported in numerous cell types [8–10]. Some IAV strains have also been implicated in manipulating autophagy, necrosis, necroptosis and pyroptosis [11–13]. Indeed, cell death caused by viral infection may involve a mixed pattern of cell death, not a certain single mode. In addition to those have been reported, IAV may also induce ferroptosis, as suggested by our proteomic analysis. We showed that SIV-induced ferroptosis depends on labile iron accumulation, ROS release as well as lipid peroxidation, thus, fits the major criteria for ferroptosis. Furthermore, we found that blocking the ferroptotic pathway using ferrostatin-1 (Fer-1) treatment suppressed ferroptosis and subsequently modulated SIV replication. Together, this study revealed a novel mode of cell death induced by IAV infection, which might improve our understanding of the underlying mechanism involved in the interaction between viruses and host cells. Additionally, ferroptosis was identified as a component of the host response to SIV infection that benefited the virus.

## Materials and methods

### Cells and viruses

The H1N1 SIV A/swine/Shanghai/3/2014 (SH/2014) strain was isolated in our laboratory from pigs with clinical symptoms of swine influenza. A549 cells were obtained from ATCC (Manassas, VA, USA) and maintained in Dulbecco’s modified Eagle’s medium (DMEM) (Gibco, Grand Island, NY, USA) supplemented with 10% fetal bovine serum (FBS) (Invitrogen, Carlsbad, CA, USA) at 37 °C with 5% CO_2_.

### Sample preparation for proteomic analysis, protein isolation and iTRAQ reagent labeling

A549 cells infected with SIV strain SH/2014 at a multiplicity of infection (MOI) of 1 or uninfected cells were collected at 24 h post-infection (h.p.i.). The cells were lysed with 400 µL of lysis buffer (100 mM NH4HCO3 (pH 8), 6 M urea and 0.2% SDS) followed by ultrasonication on ice for 10 min. The supernatant was collected and reduced with 10 mM DTT at 56 °C for 1 h and subsequently alkylated with sufficient iodoacetamide for 1 h. The extracted proteins were precipitated with precooled acetone, washed twice, and dissolved in dissolution buffer containing 0.1 M triethylammonium bicarbonate (TEAB) and 8 M urea (pH 8.5). The protein concentration was quantified with a Bradford protein assay kit (Thermo Fisher Scientific, Waltham, MA, USA). 100 μg of each protein sample was digested with Trypsin Gold (Promega, Madison, WI, USA) at 37 °C overnight and then labeled with different iTRAQ tags (iTRAQ® Reagent-8PLEX Multiplex Kit, Sigma–Aldrich, St. Louis, MO, USA). The SIV-infected samples were labeled with iTRAQ 113 (IT113), iTRAQ 114 (IT114), or iTRAQ 115 (IT115), and the mock-infected samples were labeled with iTRAQ 116 (IT116), iTRAQ 117 (IT117), or iTRAQ 118 (118). The labeled samples were then mixed with shaking for 2 h at room temperature (RT). The reaction was stopped by adding 100 μL of 50 mM Tris–HCl (pH = 8). All labeled samples were mixed to be of equal volume, desalted and lyophilized.

### HPLC fractionation

The iTRAQ-labeled peptide mixtures were fractionated using a Waters BEH C18 column (5 μm, 4.6 × 250 mm) with a Rigol L3000 HPLC system. The column oven temperature was set to 50 °C. Mobile phase A (2% acetonitrile adjusted to pH 10.0 using ammonium hydroxide) and mobile phase B (98% acetonitrile adjusted to pH 10.0 using ammonium hydroxide) were used to develop a gradient elution. The solvent gradient was set as follows: 3% B, 5 min; 3–8% B, 0.1 min; 8–18% B, 11.9 min; 18–32% B, 11 min; 32–45% B, 7 min; 45–80% B, 3 min; 80% B, 5 min; 80–5%, 0.1 min; and 5% B, 6.9 min. The elutes were collected every minute in a tube, and 10 fractions were pooled. All fractions were vacuum-dried and reconstituted in 0.1% (v/v) aqueous formic acid (FA) for subsequent LC–MS/MS analysis.

### LC–MS/MS analysis

The LC–MS/MS analysis was performed by Novogene Bioinformatics Technology Co. Ltd. The fractionated peptides were analyzed with an EASY-nLCTM 1200 UHPLC system (Thermo Fisher Scientific) coupled to a Q Exactive HF-X mass spectrometer (Thermo Fisher Scientific) operating in data-dependent acquisition (DDA) mode. During data acquisition, the mass spectrometer was operated in positive polarity mode with a spray voltage of 2.3 kV and a capillary temperature of 320 °C. The full MS scan resolution was set to 60,000 (at 200 m/z) with an (automatic gain control (AGC) target value of 3 × 10^6^ for a scan range of 350–1500 m/z. Forty precursors of with the highest abundances in the full scan were fragmented for higher-energy collision dissociation and subjected to MS/MS analysis with the following parameters: resolution, 15,000 (at m/z 200); AGC target value, 5 × 10^4^; the maximum ion injection time, 45 ms; normalized collision energy, 32%; the intensity threshold, 2.2 × 10^4^; the dynamic exclusion parameter, 60 s.

### Proteomics data normalization and analysis

The MS raw data files obtained with an Q-Exactive HF-X mass spectrometer were searched with Proteome Discoverer 2.2 search engine (PD 2.2; Thermo Fisher Scientific) against the UniProt database (homo_sapiens_uniprot_2019.01.18.fasta, containing 169,425 sequences). To reduce the probability of false peptide identification, only peptides identified at the 99% confidence interval through a Proteome Discoverer probability analysis were counted. For protein quantitation, each confident protein identification contained at least one unique peptide, and the peptide false discovery rate (FDR) was no more than 1.0%. The protein quantitation results were statistically analyzed by Mann–Whitney Tests. Only a protein with a fold change ≥ 1.5 or ≤ 0.67 and a *P* < 0.05 was considered a significantly differentially expressed proteins (DEP).

### Bioinformatic analysis

Functional classification of DEPs was performed using a Gene Ontology (GO) enrichment analysis (http://www.geneontology.org/). Pathway enrichment analysis of DEPs was carried out using the Kyoto Encyclopedia of Genes and Genomes (KEGG) database (https://www.genome.jp/kegg/). The significance of a KEGG enrichment pathway with a Bonferroni corrected *P* < 0.05 (*q* value) was determined using a hypergeometric test.

### Cell viability assay

Cell viability was determined using cell counting kit-8 reagent (CCK-8, Beyotime Biotechnology, Shanghai, China) according to the manufacturer’s protocol. Briefly, A549 cells were seeded in 96-well culture plates at a density of 1 × 10^4^ cells per well and cultured for 24 h at 37 °C. The cells were then infected with SIV at an MOI of 0.01, 0.1, or 1 or treated with 10 μM erastin (Selleck Chemicals, Houston, TX, USA) as a positive control. Cells were treated with or without the ferroptosis inhibitor Fer-1 (1 μM, Selleck Chemicals) 1 h before SIV infection to determine the role of ferroptosis in SIV-infected cells. After culture for 24 h, 10 μL of the CCK-8 reaction mixture were added to each well and incubated at 37 °C for 4 h. The absorbance of each well was measured at 450 nm with a microplate reader (Thermo Fisher Scientific), and the corresponding optical density ratio was expressed as cell viability.

### Cell morphology

For the analysis of cell morphology, A549 cells were seeded in 6-well plates and infected with SIV at an MOI of 1 or treated with 10 μM erastin for 24 h. Fer-1 (1 μM) was added 1 h before SIV infection to inhibit cell ferroptosis. Afterward, cells were fixed with 4% paraformaldehyde for 20 min at RT and washed with PBS. Images were captured using a Carl Zeiss (Oberkochen, Germany) converted fluorescence microscope (20 × objective).

### Measurement of cellular iron level

The intracellular Fe^2+^ levels were detected using the iron assay kit (Abcam, Cambridge, UK). Briefly, after treatment of the different groups, cells were washed with cold PBS and homogenized in iron assay buffer for 30 min at 37 °C. Sample was then added with iron probe and incubated at 37 °C for 60 min. The absorbance was read at 593 nm. The BCA protein determination method was used for total protein quantification, and the cellular iron concentration is presented as μM/mg protein. The intracellular Fe^2+^ levels were also detected using FerroOrange probes (Dojindo, Japan) following the manufacturer’s instructions, and Fe^2+^ image of the living cells was performed using a fluorescence microscope.

### ROS measurement

Changes in intracellular ROS levels were using DCFDA/H2DCFDA in cellular ROS assay kits (Abcam). Briefly, A549 cells were cultured in 6-well plates at a density of 2 × 10^5^ cells per well and allowed to attach overnight. After treatment, cells in groups were labeled with 20 µM DCFDA and incubated for 30 min at 37 °C. Stained cells were washed with PBS and transferred to FACS tubes. The fluorescence of each probe was detected using a CytoFLEX flow cytometer (Beckman Coulter, Suzhou, China) and analyzed by FlowJo software.

### Lipid peroxidation assay

Lipid peroxidation was determined by measuring the malondialdehyde (MDA) level with a lipid peroxidation MDA assay kit (Beyotime Biotechnology). Briefly, cells were homogenized and the supernatant was mixed with thiobarbituric acid (TBA)-glacial acetic acid reagent. Then, the TBA-MDA mixture was heated at 100 °C for 1 h. The absorbance was measured at 532 nm with a microplate reader and the MDA concentration was calculated as μM/mg protein. Lipid peroxidation was also confirmed by fluorescence observations using Liperfluo (Dojindo, Kumamoto, Japan) according to the manufacturer’s instructions. After an incubation with the indicated treatments, cells were stained with 5 μmol/L Liperfluo and Hoechst nuclear stain and then visualized under a florescence microscope (Carl Zeiss, Oberkochen, Germany).

### Reductive GSH content and NADP.^+^/NADPH assay

The intracellular levels of reductive GSH and NADP^+^/NADPH were determined using a GSSG/GSH quantification kit (Dojindo) and an NADP^+^/NADPH assay kit (Beyotime Biotechnology) respectively, following the manufacturer's instructions.

### Western blotting

A549 cells were infected with SIV at an MOI of 1 for the indicated times or treated with 1 μM Fer-1 1 h before SIV infection. The cells were collected and lysed in RIPA lysis buffer (Beyotime), denatured and loaded on 10% gels for SDS–polyacrylamide gel electrophoresis. The protein bands were then transferred onto 0.2-μm nitrocellulose membranes (Millipore, Billerica, MA, USA), blocked with 5% nonfat milk, and incubated overnight at 4 °C with primary antibodies against SLC7A11 (Cell Signaling Technology), SLC3A2L (Abcam), GPX4 (Santa Cruz), transferrin (TF) (Abcam), transferrin receptor (TFRC) (Abcam), NS1 protein (prepared in our lab) and β-actin (Cell Signaling Technology). Membranes were washed and incubated with horseradish peroxidase (HRP)-conjugated secondary antibodies (Cell Signaling Technology) for 1 h. The antibody-labeled proteins were detected by chemiluminescence using SuperSignal West Pico PLUS Chemiluminescence Substrate (Thermo Fisher Scientific) with an Amersham Imager 600 (Cytiva Sweden AB, America). Densitometry analysis was performed using the ImageJ software.

### Quantitative real-time PCR (qRT–PCR)

To explore the function of ferroptosis inhibitor treatment on SIV-induced inflammatory cytokine expression, A549 cells were treated with or without Fer-1 before SIV infection and then subjected to qRT–PCR analyses to measure the mRNA levels of IL-6, IL-8, IL-1β and TNF-α at 24 h.p.i.. Total RNA was extracted using TRIzol reagent (Invitrogen) following the manufacturer’s instructions. cDNA synthesis was performed using a SuperScript III kit (Invitrogen) and oligo-dT primer. 50 ng of the cDNA product was used as the template for qRT–PCR in a final volume of 10 μl containing SYBR Premix Ex Taq II (TaKaRa, Dalian, China). qRT–PCR was performed on a 7500 Real Time PCR System apparatus (ABI, Madison, WI, USA). The amplification conditions consisted of 95 °C for 30 s and 40 cycles of 95 °C for 5 s, 60 °C for 30 s, and 95 °C for 15 s. The changes in the levels of target mRNAs are presented as fold changes and were calculated using the comparative Ct method as previously described. The following primer sequences were used for amplification:

- IL-6 (F:AAGCCAGAGCTGTGCAGATGAGTA, R:TGTCCTGCAGCCACTGGTTC), IL-8 (F:TTTCAGAGACAGCAGAGCACA, R:CACACAGAGCTGCAGAAATCAG), IL-1β (F:GCTGATGGCCCTAAACAGATGA, R:TCCATGGCCACAACAACTGAC), TNF-α (F:CTCAGCAAGGACAGCAGAGG, R:ATGTGGCGTCTGAGGGTTGTT), GAPDH (F:GCACCGTCAAGGCTGAGAAC, R:TGGTGAAGACGCCAGTGGA).

### Titration of viruses

A549 cells grown in 96-well plates were treated with Fer-1 and then infected with SIV at an MOI of 1. The cells were then incubated for 24 h. The supernatant of infected culture was harvested, serially diluted and titrated in MDCK cells. Virus titers were calculated by the Reed-Muench method and were expressed as TCID_50_ per milliliter of supernatant.

### Statistical analysis

GraphPad Prism 6 software (GraphPad Software Inc., San Diego, CA, USA) was used for the data analyses and graph creation. All the data are presented as the means ± standard deviations (SD). The significance was determined with either unpaired two-tailed independent Student’s t test for comparisons between two groups or one-way ANOVA. A *p* < 0.05 was considered to be statistically significant.

## Results

### Determination of the protein profile using iTRAQ labeling combined with LC–MS/MS analysis

**Fig. 1:**
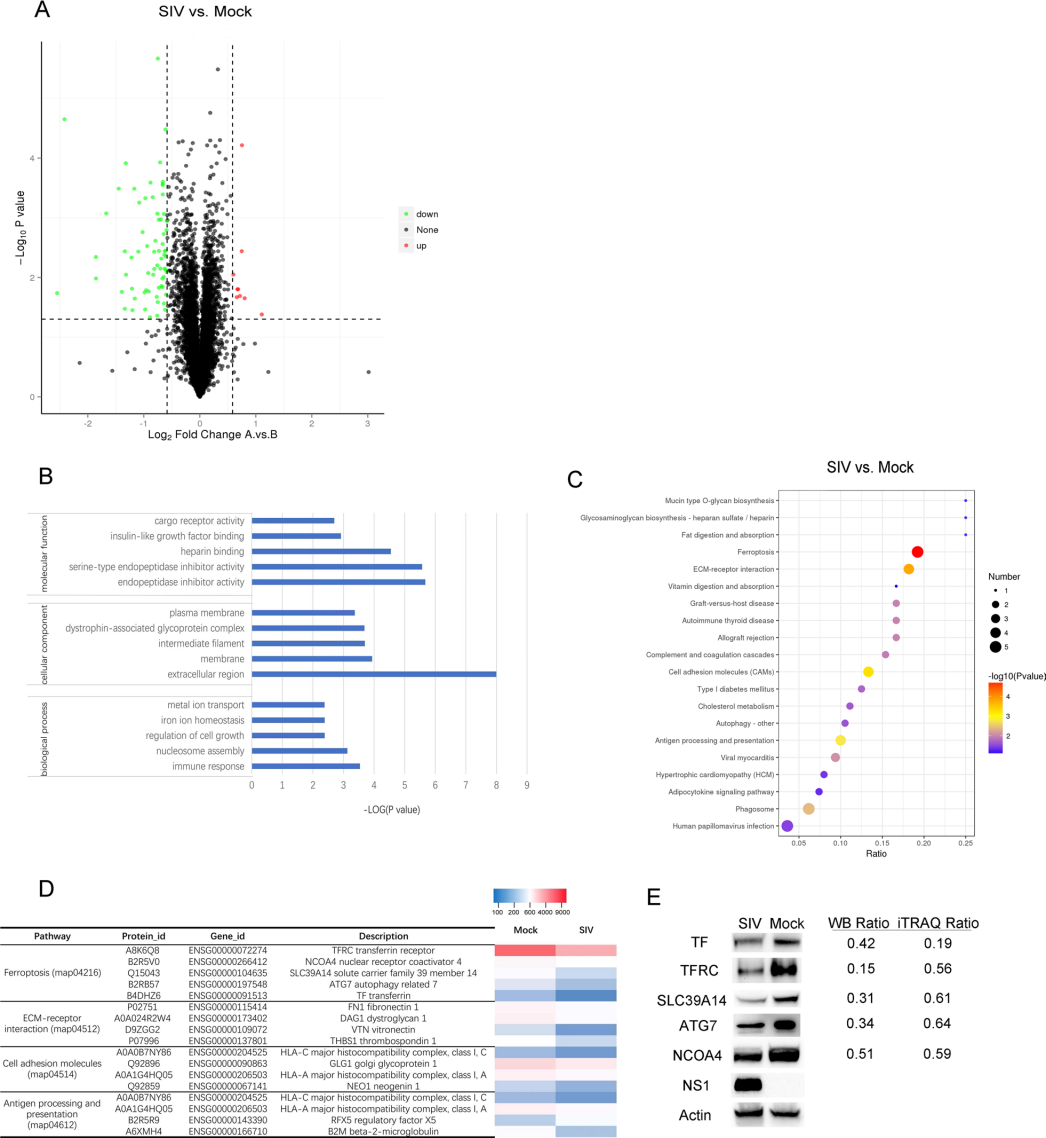
Overview of the differentially expressed proteins (DEPs) in SIV-infected cells. **A** Volcano plots of the DEPs in SIV-infected cells. Fold-changes ≥ 1.5 or ≤ 0.67 and *P* < 0.05 were considered statistically significant. Red dots, significantly upregulated proteins; green dots, significantly downregulated proteins. **B** The top five significantly enriched GO terms of ‘cellular component’, ‘biological process’ and ‘molecular function’ in SIV-infected cells. **C** KEGG pathway enrichment analysis of DEPs. **D** Abundance patterns of DEPs in four significantly enriched pathways in SIV-infected cells. **E** DEPs (TF, TFRC, SLC39A14, ATG7, and NCOA4) expression in the ferroptosis pathway of SIV-infected or mock-infected cells. The western-blot ratio and iTRAQ ratio (SIV infection group/mock group) are shown on the right

The proteome profiles of mock- and SIV- infected cells were compared after iTRAQ labeling coupled with LC–MS/MS analysis. In A549 cells infected with SIV strain SH-2014, a total of 5874 proteins were identified and quantified. A volcano plot analysis was constructed and analyzed using the criteria a fold-change ≥ 1.5 or ≤ 0.67 in protein expression and *P* ≤ 0.05 between mock- and SIV-infected cells (Fig. 1A). Based on these thresholds, 79 DEPs were identified in SIV-infected cells (Additional file 1: Table S1). The expression of 9 DEPs was upregulated, and that of 70 DEPs was significantly downregulated.

### Bioinformatics analysis of the proteome of SIV-infected cells

Based on GO enrichment analysis, all identified DEPs were assigned to three GO term categories: “cellular component”, “biological process” or “molecular function” (Additional file 2: Table S2). The five terms most significantly enriched with DEPs in SIV-infected cells are presented for each GO term category in Fig. 1B. Notably, in the biological process category, the “metal ion transport” (3 DEPs) and “iron ion homeostasis” (2 DEPs) annotations were discovered for the SIV-infected cells, indicating that intracellular iron transport and homeostasis are altered during SIV infection. Overall, GO annotations provide a comprehensive overview of the molecular characterization of SIV-infected cells. In addition, the KEGG pathway enrichment analysis showed that four metabolic pathways (*q* < 0.05) were enriched after SIV infection (Fig. 1D), of which ferroptosis was the most significantly enriched pathway (*q* = 0.0016, < 0.01). Besides, DEPs including TFRC, TF, NCOA4, SLC39A14 and ATG7 in the ferroptosis pathway showed decreased expression.

To confirm the expression levels of the DEPs in the ferroptosis pathway during SIV infection, five DEPs (TFRC, TF, NCOA4, SLC39A14 and ATG7) were assessed by western blot analysis. The results showed that the expression of TFRC, TF, NCOA4, SLC39A14 and ATG7 was decreased to some extent after SIV infection at 24 h.p.i., similar to the results obtained using the iTRAQ approach (Fig. 1E).

### SIV infection triggers the ferroptosis in A549 cells

**Fig. 2:**
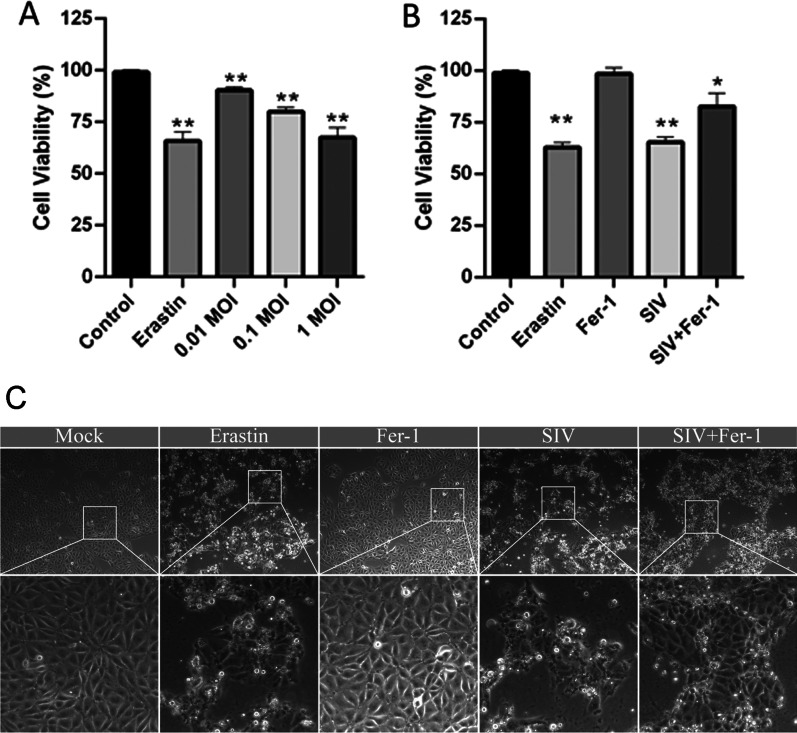
Changes in the viability of A549 cells in response to SIV. **A** A549 cells were infected of SIV at an MOI of 0.01, 0.1, 1 MOI or treated with erastin (10 μM) for 24 h, and cell viability was measured using the CCK-8 method. **B** A549 cells were treated with Fer-1 (1 μM) or not 1 h before infection with SIV (MOI of 1), and cell viability was measured at 24 h.p.i.. Erastin (10 μM) was used as the positive control, and Fer-1 (1 μM) was used as the ferroptosis inhibitor. **C** Morphological changes in cells were observed after treatment with or without Fer-1 (1 μM) 1 h before cells were infected with SIV (MOI of 1). Images were captured at a magnification of 20 × 24 h after treatment by microscopy. The error bars represent the SD of the means on the basis of three independent experiments. (*p < 0.05, **p < 0.01)

To confirm that ferroptosis is triggered after SIV infection, A549 cells were infected with SIV at an MOI of 0.01, 0.1 or 1 for 24 h. The ferroptosis inducer, erastin, was used as the positive control. Cell viability analysis showed that SIV infection significantly decreased cell viability in a dose-dependent manner. As shown in Fig. 2A, the viability of A549 cells infected with of SIV at an MOI of 0.01, 0.1 or 1 was 90.4 ± 1.6%, 80.1 ± 2.0% and 67.5 ± 4.7%, respectively, at 24 h.p.i.. Compared to the cells infected with SIV alone, the viability of SIV-infected cells treated with the ferroptosis inhibitor Fer-1 are up approximately 26.5%, indicating that decrease in cell viability caused by SIV can be mitigated by ferroptosis inhibitors(Fig. 2B). Moreover, the morphology of the SIV-infected cells was characterized by cytological changes, including cell shrinkage without nucleus rupture, and death. However, a normal morphology was partially restored by Fer-1 treatment (Fig. 2C). Thus, a ferroptosis inhibitor blocked the SIV-induced cell death of A549 cells, indicating that SIV infection may lead to cell death through ferroptosis.

### SIV-induced ferroptosis is associated with increased intracellular iron levels and oxidative stress

**Fig. 3:**
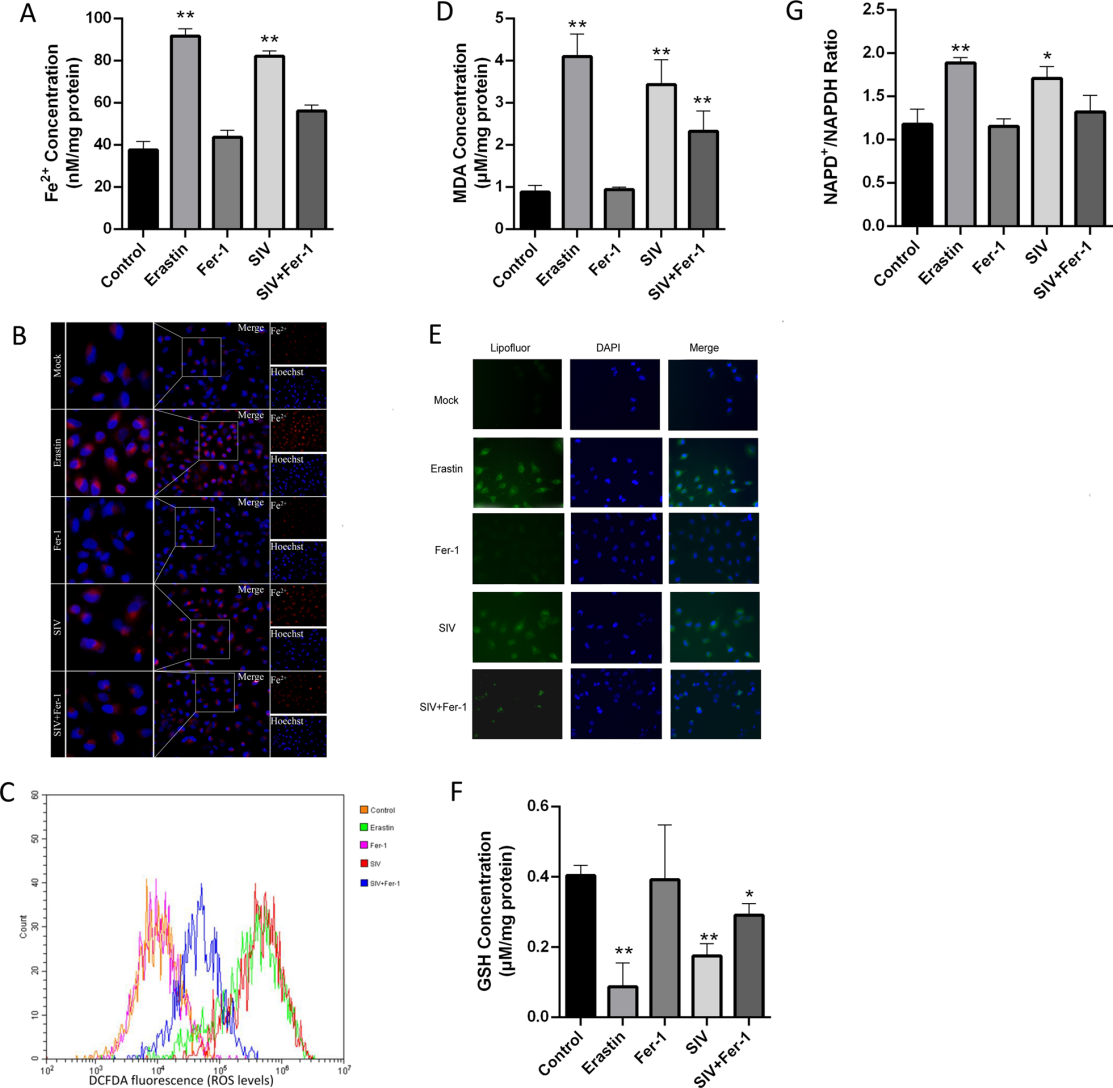
SIV induces cell death through ferroptosis. A549 cells were treated with or without 1 μM Fer-1 1 h before infection with SIV at an MOI of 1. The **A**, **B** Fe^2+^ concentration, **C** ROS level, **D** MDA concentration, **E** lipid peroxidation, **F** reduced GSH content, and **G** NADP + /NADPH ratio were determined. The error bars represent the SD of the means from three independent experiments. (*p < 0.05, **p < 0.01)

Since ferroptosis is mainly characterized by intracellular iron overload and redox imbalance, we first measured the intracellular Fe^2+^ level with an iron assay kit. As expected, SIV infection significantly increased the Fe^2+^ level in living cells, and Fer-1 treatment of these cells inhibited SIV-induced Fe^2+^ levels to some extent (Fig. 3A, B). Intracellular iron Overload can results in excessive ROS generation via the Fenton reaction and, thus, to oxidative stress and lipid peroxidation, ultimately inducing ferroptosis. We examined cellular ROS levels by staining with a DCFDA probe followed by flow cytometry and observed an increased prevalence of ROS accumulation in SIV-infected and erastin-treated cells, while Fer-1 treatment decreased this SIV-induced ROS generation (Fig. 3B). MDA is a product of lipid peroxidation. SIV infection increased MDA levels in A549 cells, but it significantly reduced in cells by treated with Fer-1 before SIV infection (Fig. 3C). Lipid peroxidation was also detected using Liperfluo staining, and the fluorescence signal was found increased in SIV-infected cells, confirming that SIV induced lipid peroxidation in A549 cells (Fig. 3D). As an important antioxidant, GSH is an important factor in determining antioxidant capacity. Therefore, we examined changes in the GSH level upon SIV infection. The results showed that SIV infection led to a remarkable depletion of GSH compared with the control, and this decrease in GSH levels impaired cellular antioxidant defenses. As a coenzyme of GSH reductase, NADPH maintains the GSH content in cells. As shown in Fig. 3F, decreased levels of NADPH were observed in response to SIV infection. Consistently, the reduction in GSH and NADPH levels in SIV-infected cells was also mitigated by Fer-1. Taken together, SIV infection induces iron overload, ROS accumulation and lipid peroxidation through an iron-dependent mechanism, consistent with ferroptosis.

### System Xc.^−^/GPX4 suppression and transferrin disorder contribute to SIV induced ferroptosis

**Fig. 4:**
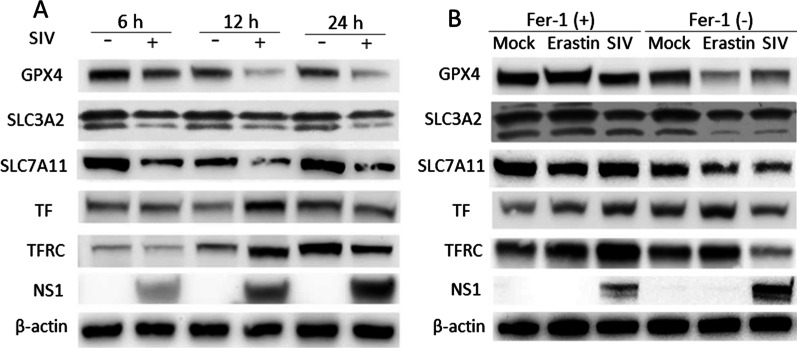
SIV induces ferroptosis by suppressing the system Xc^−^/GPX4 axis. **A** A549 cells were infected with SIV at an MOI of 1. At 6, 12, and 24 h.p.i., the expression of GPX4, SLC7A11, SLC3A2, TF, and TFRC was determined by western blot. β-actin was the loading control. **B** A549 cells were treated with Fer-1 (1 μM), Lip-1 (200 μM) or not followed by infection with SIV at an MOI of 1. At 24 h, the expression of GPX4, SLC7A11, SLC3A2, TF, and TFRC was determined by western blot

As ferroptosis is caused by iron-dependent lipid peroxidation, and transferrin-mediated iron transport into cells is the most common iron uptake pathway, we hypothesized that SIV infection results in an imbalance in iron metabolism in cells. As shown in Fig. 4A, TF and TFRC expression was significantly increased in SIV-treated cells at 12 h.p.i., which is consistent with the high iron content in the SIV infection group. However, the expression of TF and TFRC was decreased at 24 h.p.i., and it may affect iron transport and eventually lead to iron disorder in cells. The system Xc^−^/GPX4 axis suppresses lipid peroxidation and is a vital pathway involved in inhibiting ferroptosis. In the current study, SIV infection suppressed the expression of the two system Xc^−^ subunits SLC3A2 and SLC7A11 in a time-dependent manner, and GPX4 expression was simultaneously downregulated. Additionally, downregulation of SLC7A11, SLC3A2 and GPX4 expression by SIV infection was partially rescued by Fer-1 treatment, confirming that SIV induced ferroptosis by inhibiting the system Xc^−^/GPX4 axis (Fig. 4B). Collectively, our results demonstrated that SIV infection causes intercellular iron disorder, inhibits system Xc^−^/GPX4 axis activation, and subsequently promotes cell lipid peroxidation and ferroptosis.

### Ferroptosis induced by SIV is critical for virus replication and inflammatory responses in A549 cells

**Fig. 5:**
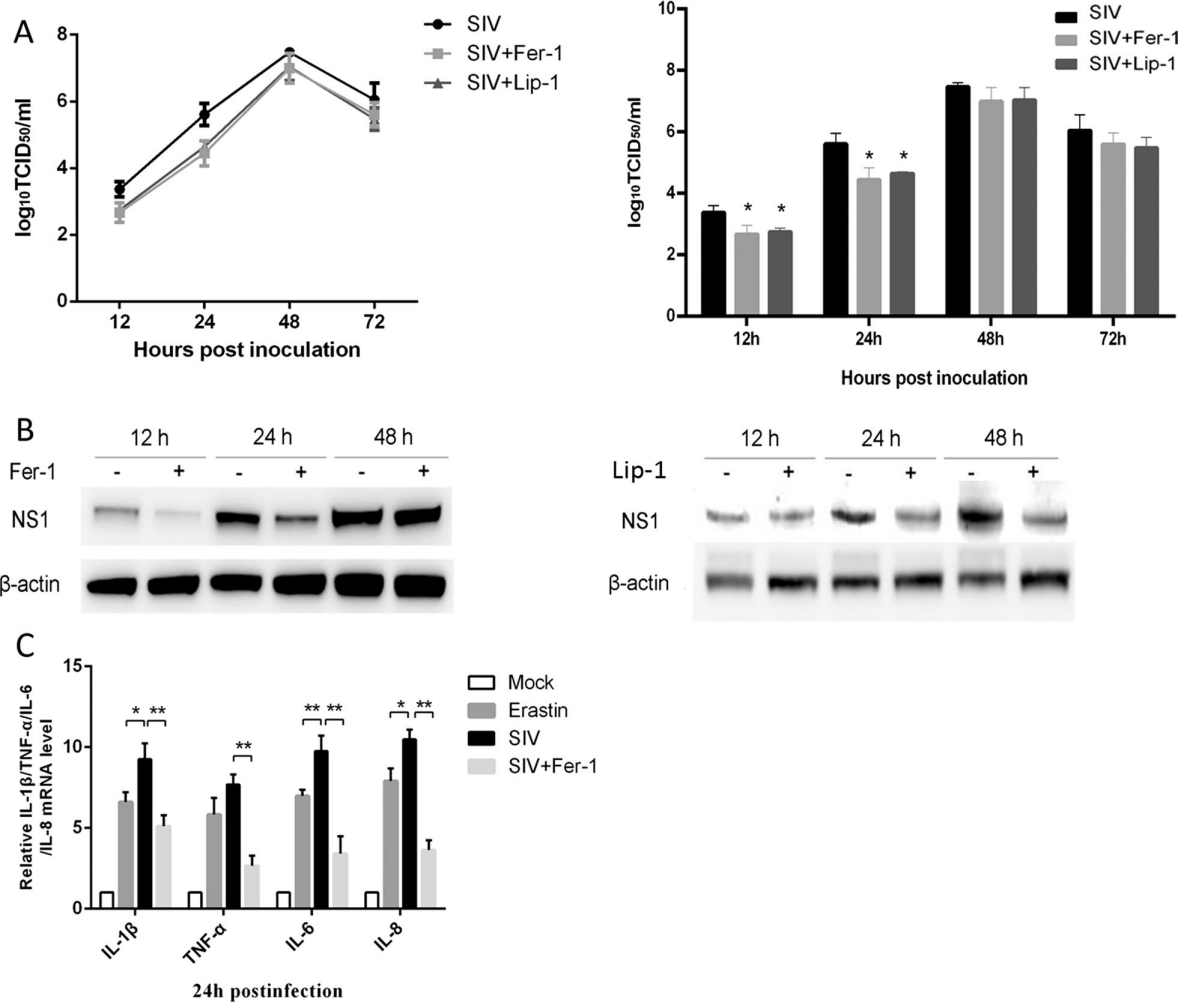
Effect of ferroptosis inhibitor on SIV replication and SIV-induced cytokine expression. A549 cells were treated with Fer-1 (1 μM) or not followed by infection with SIV at an MOI of 1. **A** Viral growth in A549 cells is reported as the TCID_50_ value 12, 24, 48 and 72 h.p.i.. **B** Cell lysates were collected at 12, 24 and 72 h.p.i. for western blot analysis of SIV NS1 protein levels. **C** IL-1β, TNF-α, IL-6, and IL-8 mRNA levels in the cell lysates were quantified by qRT–PCR. The error bars represent the SD of the means on the basis of three independent experiments (*p < 0.05, **p < 0.01)

To measure the effect of ferroptosis on SIV infection, SIV-infected cells were treated with or without Fer-1, the supernatant was collected, and the progeny virus in the supernatant was detected by TCID_50_ assay at the indicated time points. The SIV titers were reduced in Fer-1 treated cells, but with the extension of SIV infection after 24 h.p.i., the effect of Fer-1 on reducing the viral titer decreased. As shown in Fig. 5A, Fer-1 treated cells presented approximately 4.7-fold lower titers at 12 h.p.i and 11-fold lower titers at 24 h.p.i., respectively, compared with untreated cells.. A western blot analysis showed that Fer-1 treatment also inhibited the expression of SIV NS1 protein, showing a reduction of approximately by 43% at 12 h.p.i. and 77% at 24 h.p.i., but no differences in NS1 protein expression were observed between the two groups at 48 h.p.i.. (Fig. 5B). We also used another ferroptosis inhibitor, liproxstatin-1(Lip-1) to exclude the specific actions of Fer-1, and we got the similar results to the Fer-1 treatment. Moreover, we investigated the effect of SIV-induced ferroptosis on inflammatory cytokine production. As shown in Fig. 5C, both erastin treated cells and SIV-infected cells exhibited higher expression of the IL-1β, TNF-α, IL-6, and IL-8 mRNAs at 24 h.p.i. and the expression of IL-1β, TNF-α, IL-6, and IL-8 was much more strongly induced by SIV infection. In SIV-infected cells treated with Fer-1, inflammatory cytokine production was significantly impaired, although it was still higher than that in the mock groups. These data demonstrated that inhibition of ferroptosis exerts anti-inflammatory effects during SIV infection.

## Discussion

As a form of iron-dependent cell death, ferroptosis is characterized by intracellular iron overload and lipid peroxidation. Recently, IAV has been reported to induce several programmed cell death pathways, such as apoptosis, cell necrosis, necroptosis and pyroptosis, which play essential roles in the host defense against IAV infection. In this study, iTRAQ labing combined with LC–MS/MS proteomic was used to profile protein expression in A549 cells infected with H1N1 SIV. Several proteins closely relevant to iron homeostasis and transport were identified and the KEGG pathway enrichment analysis showed that the ferroptosis signaling pathway was highly enriched in response to SIV infection. After considering these data, we aimed to determine whether SIV infection can induce a newly discovered cell death form, ferroptosis, and the potential mechanisms involved.

Iron is involved in regulating many important biological processes, including redox reactions, cell proliferation, the cell cycle, and DNA synthesis. Therefore, iron homeostasis needs to be maintained in the cellular environment. Intracellular iron uptake mainly depends on TFRC, a carrier protein, that binds TF at the cell surface, thereby controlling intracellular iron levels [14]. Our results showed that TF and TFRC expression in A549 cells was increased 6 and 12 h.p.i., but their levels were unexpectedly downregulated at 24 h.p.i.. The results showed the excessive iron uptake and storage were induced by SIV infection, and downregulated expression of carrier proteins TF and TFRC can be interpreted as a compensation mechanism for defensive measures. We hypothesize that supplemental feedback is activated to initiate protective measures against uptake of excessive iron. Pathological labile iron disorder leads to rapid ROS accumulation, which is essential for ferroptosis. The data reported herein suggested that iron accumulates and the expression of iron-binding proteins is altered after SIV infection, facilitating A549 cell ferroptosis.

ROS accumulation has been measured in infections with many different viruses, and the failure to maintain a normal cellular redox state contributes to viral pathogenesis through the substantial induction of cell death [15]. A previous study confirmed that ROS are strongly associated with a variety of cell death pathways including necroptosis, ferroptosis and autophagic cell death [16]. In dengue virus (DENV), for instance, ROS are essential for driving mitochondrial apoptosis of infected monocyte-derived dendritic cells and stimulate innate immune responses [17]. You et al. revealed a mechanism by which enterovirus 71 induces apoptosis and autophagy by promoting ROS generation in neural cells [18]. ROS production has also been reported to be required for activation of macroautophagy/autophagy during IAV infection [19]. In the present study, we found that ferroptosis inhibitor reversed the effects of SIV infection on iron overload and ROS release. Consistently, MDA production, GSH and NADPH depletion associated with the response to SIV infection were all mitigated by treatment with ferroptosis inhibitor. System Xc^−^ is an amino acid antiporter that mediates cystine import for the synthesis of the major antioxidant GSH. It consists of a light-chain subunit xCT (SLC7A11) and a heavy-chain subunit CD98 (SLC3A2) [20, 21]. Inhibition of system Xc^−^ impairs GSH production and inactivation of the phospholipid peroxidase GPX4, eventually resulting in overwhelming lipid peroxidation that causes cell death [22]. In the present study, SLC7A11, SLC3A2L and GPX4 levels were all reduced in SIV-infected cells and this effect was partially reversed by Fer-1, supporting that SIV infection regulate system Xc^−^/GPX4 axis to improve ferroptosis.

Recently, ferroptosis has been suggested to induce autophagy. Park et al. confirmed that autophagy was triggered by erastin-induced ROS in ferroptosis [23]. They found that erastin-induced ferroptotic cell death was clearly inhibited in autophagy-deficient cells, suggesting that ferroptosis is an autophagic cell death process. Similarly, Gao et al. documented that ferroptosis initiation activates autophagy to regulate cellular iron homeostasis and cellular ROS generation. In particular, autophagy leads to the degradation of the cellular iron stock protein ferritin and cause an increase in cellular labile iron levels via the NCOA4-mediated autophagy pathway, termed ferritinophagy [24]. NCOA4 is a selective cargo receptor that mediates the autophagic degradation of ferritin in ferroptosis. In our bioinformatics and immunoblotting analyses, NCOA4 was significantly downregulated by SIV infection at 24 h.p.i.. Additionally, the level of ATG 7, which is critical for the formation of autophagosomes, decreased within 24 h.p.i.. The downregulation of ATG7 and NCOA4 expression may have been due to their binding to ferritin in autophagosomes and their subsequent delivery into lysosomes for degradation, thereby releasing iron through the autophagic process [25, 26]. Thus, autophagy is accompanied by ferroptosis and these processes are related to one another somehow. Functional characterization of the ATG7-NCOA4 autophagy pathway in SIV-induced ferroptosis needs further study.

IAV infections lead to inflammatory cytokine secretion or even excessive inflammatory reactions known as the cytokine storm. Intracellular pattern recognition receptors (PRRs) such as retinoic acid-inducible gene I (RIG-I) or Toll-like receptors, typically recognize viral RNA and trigger inflammation [27]. Recently study showed that ferroptosis affects inflammation through immunogenicity, and excessive iron is detrimental to the redox balance and can further enhance the production of inflammatory factors [28]. In studies of secondary brain injury (SBI), Zhang et al. reported that ferroptosis inhibitor could ameliorate brain injury. They found that inhibiting ferroptosis with Fer-1 significantly reduced the levels of ROS and certain inflammatory factors, such as IL-1β and TNFα, in a rat model [29]. Similar to SBI, radiation-induced lung fibrosis (RILF) is considered to involve excessive ROS-induced oxidative damage as the initiation of inflammation. For example, Li et al. showed that Lip-1 treatment blocked RILF by downregulating inflammatory factors [30]. In the present study, ferroptosis inhibitors alleviated virus-induced inflammation, as indicated by the increases in IL-1β, TNF-α, IL-6, and IL-8 levels caused by SIV infection, which were abrogated by Fer-1 treatment. In summary, ferroptosis inhibitors have shown significant benefits when applied to certain diseases because of their anti-inflammatory effects and may be potential new therapeutic treatments that protect cells from the dysregulated cytokine production induced by SIV infection.

## Conclusions

In summary, SIV infection disrupts intracellular iron and redox homeostasis. Meanwhile, SIV infection inhibits the system Xc^−^/GPX4 axis, which contributes to overwhelming lipid peroxidation, subsequently leading to cell ferroptosis. In addition, the downregulation of ATG 7 and NCOA4 may imply that autophagy is accompanied by ferroptosis during SIV infection.

## Supplementary Information

**Additional file 1:** The fold changes of all significantly differently expressed proteins  in SIV-infected cells.

**Additional file 2: **Gene Ontology analysis of DEPs in SIV-infected cells.

## Data Availability

All data generated or analysed during this study were available from the corresponding author on reasonable request.

## Abbreviations

IAV: Influenza A virus
SIV: Swine influenza virus
ROS: Reactive oxygen species
GPX4: Glutathione peroxidase 4
GSH: Glutathione
Fer-1: Ferrostatin-1
Lip-1: Liproxstatin-1
DEPs: Differentially expressed proteins
MDA: Malondialdehyde
TF: Transferrin
TFRC: Transferrin receptor
MOI: Multiplicity of infection
h.p.i.: Hours post infection
GO: Gene ontology
KEGG: Encyclopedia of genes and genomes
